# Supplementary material for: GL5.2, a Quantitative Trait Locus for Rice Grain Shape, Encodes a RING-Type E3 Ubiquitin Ligase
Source: Plants (Basel). 2024 Sep 8;13(17):2521. doi: 10.3390/plants13172521 (PMC11397561; doi:10.3390/plants13172521)
Supplement: Supplementary file 1 [file plants-13-02521-s001.zip › plants-3171529-supplementary.pdf]

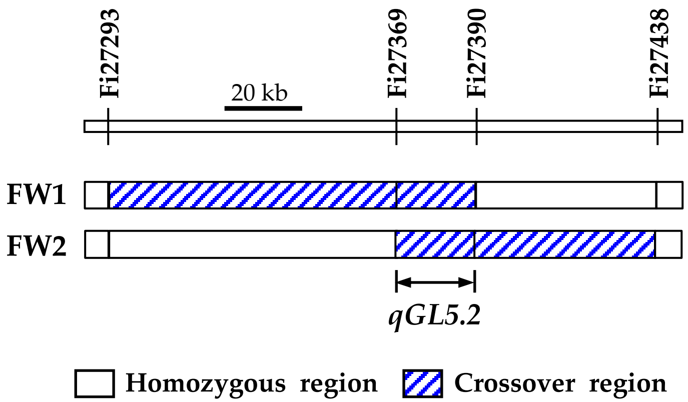

Figure S1. Segregating regions of the two near isogenic line populations.

Table S1. Haplotypes of *GL5.2* in rice germplasms.

Haplotype	SNP1	SNP2	SNP3	SNP4	SNP5	INDEL	SNP6	SNP7	SNP8	SNP9	SNP10	SNP11	SNP12	Sum	Ind	Jap	Inter	AUS	Aro
Hap1	A	G	A	A	T	T	T	T	G	T	C	T	G	1105	1082	8	11	2	2
Hap2	C	G	A	G	C	T	C	C	A	T	T	T	A	959	14	887	15	0	43
Hap3	A	G	A	A	T	T	T	T	G	T	C	T	A	775	541	184	10	35	5
Hap4	A	G	A	A	T	T	C	T	G	T	C	T	A	460	105	115	10	216	14
Hap5	A	G	A	A	T	DEL	C	T	G	T	C	T	A	294	115	169	7	0	3
Hap6	A	T	A	G	T	DEL	C	T	G	T	C	T	A	138	124	0	3	0	11
Hap7	A	T	A	G	T	T	C	T	G	T	C	T	A	86	82	0	0	0	4
Hap8	A	G	A	A	T	DEL	T	T	G	T	C	T	G	47	47	0	0	0	0
Hap9	A	G	A	A	T	DEL	T	T	G	T	C	T	A	24	12	11	0	0	1
Hap10	DEL	G	A	DEL	C	T	C	DEL	DEL	T	DEL	T	A	10	1	8	0	0	1

Ind, *indica* germplasms; Jap, *japonica* germplasms; Inter, intermediate germplasms; Aro, aromatic.

Table S2. Primers used in this study.

Name	Primer sequence	Purpose
Fi27293	F, 5'- TAAACATGCACGGGTTCCAG -3'<br>R, 5'- TTCCATCACAGGTTGTCCCA -3'	Mapping
Fi27369	F, 5'- AGTTTAAAACCCCGACTCTCC -3'<br>R, 5'- TGCACTCACCAAGTATCACG -3'	Mapping
Fi27390	F, 5'- GTACTTTTATACTACGCGATG -3'<br>R, 5'- TCTAGCTATGAATCTGAACGC -3'	Mapping
Fi27438	F, 5'- CGCGACGGAAATGATCAAGC -3'<br>R, 5'- GGAGGAGAGAGCAACCAACC -3'	Mapping
cri-1	F, 5'- TGTGTGCTTCAAAAATGAGGAAACAA -3'<br>R, 5'- AAACTTGTTTCCTCATTTTTGAAGCA -3'	Vector construction
seqcri-1	F, 5'- ACAAATCCAAATAACAGGAGCCA -3'<br>R, 5'- TCCAATAACTTCAACGGTATGAC -3'	Sequencing
Hyg	F, 5'- GTTTATCGGCACTTTGCATCG -3'<br>R, 5'- GGAGCATATACGCCCGGAGT -3'	Transgene detection
